# FAMA and bHLH090: Coordinating myrosin cell development and herbivory defense

**DOI:** 10.1093/plphys/kiaf544

**Published:** 2025-10-27

**Authors:** Marcella Teixeira

**Affiliations:** Assistant Features Editor, Plant Physiology, American Society of Plant Biologists; Department of Plant Pathology, Washington State University, Pullman, WA 99163, USA

Plants are exposed to different stresses during their life cycle. To fight off herbivores, the Brassicaceae family has developed a defense system known as “mustard oil bomb.” Under normal conditions, glucosinolates (GSLs) and myrosinase, which breaks down GSLs into compounds toxic to herbivores, are kept in separate compartments but positioned close enough to be quickly mixed (Luthy and Matile 1984; Ratzka et al. 2002; Shirakawa and Hara-Nishimura 2018). When an herbivore feeds and breaches plant tissue, the myrosinase and GSLs react and the bomb goes off, resulting in a burst of toxic metabolites that halt the herbivore attack. In a brilliant evolutionary adaptation, Brassicaceae plants show that survival often depends on timely and precise response to attack.

The “mustard oil bomb” has been well characterized in Arabidopsis. Myrosinases, encoded by Thioglucoside Glucohydrolase (TGG) genes, accumulate in specialized myrosin cells (MCs), including guard cells (GCs) and myrosin idioblast cells (MIs). Loss of 2 *TGG*s in the *tgg1 tgg2* double mutant increases herbivore susceptibility (Barth and Jander 2006). TGGI and TGG2 accumulation and MI production are regulated by the basic-helix-loop-helix (bHLH) transcription factor FAMA (Li and Sack 2014; Shirakawa et al. 2014, 2016), although its targets in MI development are yet to be determined. Interestingly, the expression of another transcription factor, *bHLH090*, is upregulated in *FAMA*-overexpressing plants (Hachez et al., 2011; Shirakawa et al. 2014).

In this issue of *Plant Physiology*, Zhou et al. (2025) investigated the roles of bHLH090 in MI development and herbivory response. First, using a *bHLH090* promoter-driven GUS reporter, the authors demonstrated spatial overlap of bHLH090 expression with the described MI distribution (Barth and Jander 2006; Shirakawa et al. 2014). Genetic analysis revealed that *bHLH090* overexpression boosts MI production and myrosinase activity, culminating in enhanced resistance to insect herbivory (Fig. 1).

**Figure 1.**
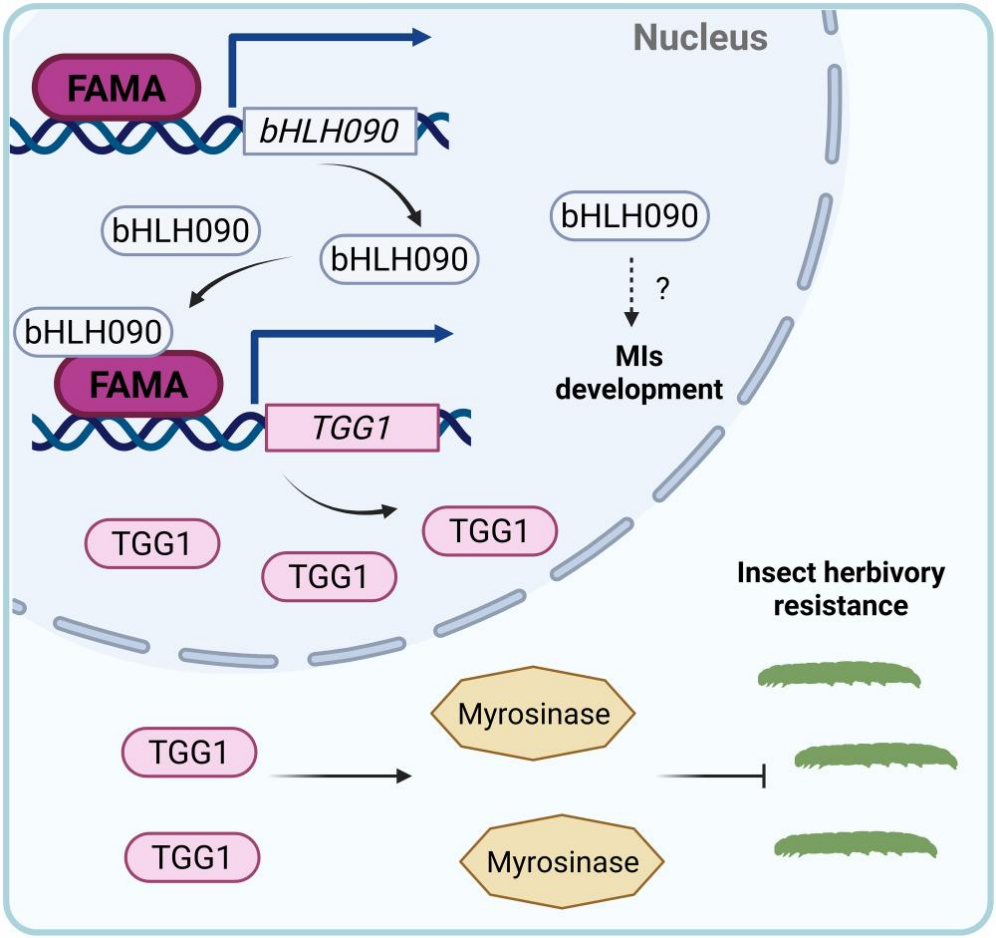
FAMA-bHLH090-TGG1 cascade: MI development and insect herbivory resistance. The bHLH transcription factor FAMA activates *bHLH090* expression in MIs. bHLH090 proteins have 2 fates: the development of MIs through yet unknown mechanisms, and interaction with FAMA to activate *TGG1* expression and further increase in Myrosinase levels, which ultimately results in increased resistance against insect herbivory (Adapted from Figure 8, Zhou et al. 2025; created in BioRender https://BioRender.com/zkltv6g).

Given FAMA's function in MI development and *bHLH090* high expression in FAMA-overexpressing lines, the authors investigated FAMA's role in bHLH090 regulation by introducing *pbHLH090:GUS* into the *fama-1* mutant. They discovered that *bHLH090* expression was reduced in *fama* mutant, suggesting that *bHLH090* expression in MIs is dependent on FAMA function. Consistently, transient expression in *Nicotiana benthamiana* showed that FAMA activated *bHLH090* expression. The authors further showed that *bHLH090* activation is a result of FAMA's direct binding to bHLH090 promoter through chromatin immunoprecipitation-qPCR and electrophoretic mobility shift assay.

If *bHLH090* is the sole target of FAMA, we would expect that overexpression of *bHLH090* in *fama-1* mutant plants would be sufficient to rescue the defects of MI development in *fama*. Interestingly, *bHLH090* overexpression in *fama-1* background could not rescue *fama-1* mutants' MI defect, suggesting that FAMA's role in MI development is more than simply activating b*HLH090* expression. Therefore, besides activation of *bHLH090* expression, FAMA is necessary for bHLH090 function. Because bHLH transcription factors have been shown to form protein complexes to regulate gene expression (Kanaoka et al. 2008), the authors hypothesized that FAMA and bHLH090 would be part of a regulatory protein complex. Consistently, FAMA and bHLH090 physically interact, as demonstrated by results from yeast 2-hybrid, pull down, and firefly luciferase complementation imaging.

Finally, because TGG1 is regulated by both FAMA and bHLH090, the authors asked if the interaction between FAMA and bHLH090 regulates *TGG1* expression. Using a transient expression assay, they showed that coexpression of FAMA and bHLH090 results in increased expression of *TGG1*, revealing a synergistic effect between the two proteins (Fig. 1).

In an orchestrated dance, Arabidopsis uses the transcription factor bHLH090 to amplify a defense response through the activation of TGG1 (Fig. 1). By enhancing or rewiring this defense switch, Brassicaceae crops can be engineered to start a rapid and localized response against herbivores, reducing dependence on chemical pesticides. On a broader perspective, understanding how plants balance the energetic cost of defense with growth can inform strategies for designing stress-resilient crops.

On the molecular mechanism's perspective, the work presented by Zhou et al. (2025) raises several questions. Which other bHLH family members might have a role in MI development? Since *bHLH090* overexpression was insufficient to rescue MI development in *fama-1*, what additional factors are involved in MI development and function? How do herbivory cues integrate with the FAMA-bHLH090-TGG1 cascade to trigger timely activation of the defense bomb?

The Zhou et al. (2025) study adds a new layer of complexity to the mustard oil bomb and opens the door to leveraging this evolutionary innovation in agriculture. The challenge now is to expand these insights beyond Arabidopsis, translating molecular choreography into major crops in field-ready strategies.

## Data Availability

No data is generated in this study.
